# Psychosocial Factors Associated with a Positive Antenatal Depression Screen: A Cross-Sectional Study of Perception of Motherhood, Attachment Style, Emotional History and Sleep Problems

**DOI:** 10.3390/diagnostics16152320

**Published:** 2026-07-24

**Authors:** Nicoleta Șoldan, Monica Petrescu, Suzana Turcu, Adriana Borosanu, Cristina Stan, Mioara Șoldan, Cristiana Glavce

**Affiliations:** 1 Department of Medical Anthropology, “Francisc I. Rainer” Institute of Anthropology, Romanian Academy, Eroii Sanitari Bd., No. 8, 050474 Bucharest, Romania; monicapetrescu20032002@yahoo.ca (M.P.); suzana.turcu@antropologia.ro (S.T.); adriana_borosanu@yahoo.com (A.B.); cristinabest2004@yahoo.com (C.S.); glavcecristiana@yahoo.fr (C.G.); 2 Independent Researcher, 014194 Bucharest, Romania; mioara.soldan@gmail.com

**Keywords:** antenatal depression, adult attachment, perception of motherhood, psychosocial vulnerability, Firth penalized regression, rare-events data, EPDS, third-trimester pregnancy

## Abstract

**Background/Objectives**: Identifying pregnant women with heightened emotional vulnerability remains a challenge in antenatal care. A positive screening result is a comparatively infrequent event in clinical samples of moderate size, which complicates the statistical modelling of associated factors. This study aimed to characterise the psychosocial profile associated with a positive antenatal depression screen, integrating attachment style, perception of motherhood, previous emotional history and sleep problems. **Methods**: A total of 140 women in the third trimester of pregnancy were assessed cross-sectionally using the Edinburgh Postnatal Depression Scale (EPDS), with a threshold of 14 or above defining a positive screen; the Adult Attachment Scale; and single-item psychosocial indicators. Associations were examined using the chi-square test, the Fisher–Freeman–Halton exact test, and Firth penalized logistic regression, chosen to correct the separation arising from the small number of events. **Results**: The screen was positive in 15.0% of participants. Negative perception of motherhood, anxious–ambivalent attachment, previous emotional history and sleep disturbances were all significantly associated with depressive symptoms; in the multivariable model, only negative perception of motherhood remained independently associated with a positive screen. **Conclusions**: Structured psychosocial assessment may usefully complement standard EPDS screening in the early identification of antenatal emotional vulnerability.

## 1. Introduction

Antenatal depression ranks among the most frequent psychological complications of pregnancy and constitutes a major public health concern. Recent systematic reviews and meta-analyses place the prevalence of depressive symptoms during the antenatal period at approximately 15–20%, with higher figures in populations exposed to socio-economic vulnerability and elevated psychological stress [1,2]. Beyond their immediate impact, antenatal depressive symptoms have been linked to a broad spectrum of adverse outcomes, including an increased risk of postpartum depression, difficulties in the mother–infant relationship, and disturbances in the child’s emotional and cognitive development [3,4]. Antenatal depression is no longer conceptualised as a transient emotional difficulty, but rather as a multifactorial condition situated at the intersection of biological, psychological and social mechanisms [5].

From this perspective, antenatal psychological screening and the early identification of emotional risk factors are regarded as priorities in obstetric practice and in contemporary public health strategies [6]. The American College of Obstetricians and Gynecologists (ACOG) recommends routine depression screening within antenatal care, embedded in clear referral and treatment pathways [7]. Validated screening instruments such as the Edinburgh Postnatal Depression Scale (EPDS) are essential in this context, and their diagnostic accuracy for perinatal depression has been confirmed in recent validation studies [8]. Symptom scores alone, however, do not capture the full extent of psychosocial vulnerability, and complementing them with relational and cognitive indicators may improve the identification of women at increased risk [9].

Attachment theory offers one of the most robust explanatory frameworks for individual differences in emotional regulation and psychological adaptation during the perinatal period. Following the perspective developed by Bowlby and subsequently extended through research on adult attachment, attachment styles reflect internal working models of interpersonal relationships that remain relatively stable across the lifespan [10,11]. During pregnancy, attachment systems become particularly active, as the transition to motherhood entails heightened needs for interpersonal security and emotional support [12]. Empirical evidence consistently indicates that insecure attachment styles—the anxious–ambivalent style in particular—are associated with an increased risk of perinatal depressive and anxiety symptoms [13,14]. Proposed mechanisms include hyperactivation of the attachment system, heightened sensitivity to rejection, and persistent difficulties in emotional regulation. Secure attachment, by contrast, is associated with adaptive coping strategies and effective use of social support, and is regarded as a protective factor for perinatal mental health [15].

A second relevant psychological dimension is the perception of motherhood. The manner in which a woman internalises the experience of pregnancy and anticipates the maternal role may substantially shape antenatal emotional adaptation. Recent studies indicate that negative or ambivalent maternal representations are associated with elevated levels of depression and anxiety during pregnancy, whereas positive representations correlate with better affective adjustment [16,17]. From this standpoint, the perception of motherhood may be conceptualised as an integrative variable, positioned at the intersection of the cognitive, affective and relational processes involved in the transition to motherhood [18].

A third dimension, often insufficiently integrated into screening models, is previous emotional history. Numerous studies have demonstrated that a history of anxiety or depression is among the most consistent factors associated with depression during pregnancy [19,20]. Emotional vulnerability persists over time and may be reactivated in contexts of major psychological demand, such as pregnancy and the transition to the parental role [21]. Including emotional history in antenatal assessment may refine the identification of women at increased risk.

Contextual and socio-demographic factors likewise shape the emotional experience of motherhood. Perceived partner support is among the most consistent protective factors identified in the perinatal mental health literature [22], while unplanned pregnancy has been associated with elevated psychological distress [23]. Sleep disturbances during pregnancy represent a further factor frequently associated with perinatal depressive symptoms, and one that is readily assessed within antenatal care [24].

In Romania, changes in family structure and the tendency to postpone motherhood to later ages reflect socio-cultural shifts documented in the national literature [25]. Family functioning—the cohesion and adaptability of the couple and the wider family—has been described through models validated in the Romanian population [26], providing a framework for understanding the relational resources relevant to adaptation during pregnancy.

Despite growing interest in perinatal mental health, the simultaneous integration of psychological, relational and cognitive factors into unified explanatory models of antenatal depression remains insufficiently developed, with most studies addressing variables in isolation. Data from Central and Eastern European countries are still limited, notwithstanding socio-cultural particularities that may influence psychological adaptation during pregnancy. A further methodological challenge, rarely addressed explicitly, arises when the outcome of interest is infrequent—as with a positive screen in a sample of moderate size—which can produce quasi-complete separation in regression models.

The aim of the present study was therefore to investigate psychosocial factors associated with a positive antenatal depression screen (EPDS ≥ 14, defined as the dependent variable), integrating several complementary psychosocial dimensions, including attachment style, perception of motherhood, previous emotional history, and sleep disturbances, in a sample of women in the third trimester of pregnancy. Specifically, it addressed two main research questions: which of these psychosocial factors were associated with a positive antenatal depression screen, and which remained independently associated in the multivariable model. To address the potential instability of estimates arising from the limited number of positive screening outcomes and the risk of quasi-complete separation, multivariable logistic regression was performed using Firth’s penalized likelihood approach.

## 2. Materials and Methods

### 2.1. Study Design and Research Setting

The present cross-sectional observational study forms part of a larger, sequentially conducted mixed-methods research project addressing the psychosocial and psychological factors involved in maternal adaptation during the antenatal period. The project was carried out in a single cohort of 140 pregnant women, assessed successively over the course of pregnancy. The first two stages of the research, conducted in the first and second trimesters, comprised anthropological assessment and qualitative analysis of the experience of motherhood, and informed the conceptual grounding and operationalisation of the psychosocial variables examined in the quantitative stage. The analysis presented here drew on data collected in the third stage of the project and examined the associations between adult attachment style, perception of motherhood, previous emotional history, sleep problems and antenatal depressive symptoms.

Data from the same sample of 140 participants have been partly reported in a previous publication [27], which examined the association of attachment style and perception of motherhood with the severity of antenatal depressive symptoms using binary logistic regression with bootstrap-estimated confidence intervals. The present study differs from that analysis both in its objective and in its methodological approach: (i) it incorporates previous emotional history and sleep problems as additional psychosocial dimensions, alongside attachment style and perception of motherhood; (ii) it explicitly addresses the quasi-complete separation generated by the small number of events, using Firth penalized logistic regression as the primary multivariable analysis; and (iii) it complements the bivariate analyses with the Fisher–Freeman–Halton exact test for contingency tables containing sparse cells. There is no text overlap between the two manuscripts, and the multivariable analyses reported here are distinct from those published previously.

The data analysed in this article were collected between November 2023 and June 2024 within obstetric and antenatal monitoring services in an urban setting in Romania. At this stage, all 140 participants were reassessed in the third trimester of pregnancy (28–39 weeks of gestation). This gestational window was selected because the third trimester is the period in which the emotional and relational adaptation processes associated with the approach of childbirth and the transition to the maternal role become particularly intense. It is therefore a relevant stage for assessing antenatal depressive symptoms and their associated psychosocial factors.

This observational study was reported in accordance with the STROBE Statement [28], and the completed STROBE checklist is provided as Supplementary Material File S1.

### 2.2. Participants

The final sample comprised 140 pregnant women in the third trimester of pregnancy (28–39 weeks of gestation), aged between 28 and 40 years (mean 33.56 ± 3.19 years). Of these, 86 (61.4%) were between 28 and 32 weeks of gestation (early third trimester) and 54 (38.6%) between 33 and 39 weeks (late third trimester). All eligible women who agreed to take part were enrolled consecutively, without further selection procedures, in order to reduce the risk of selection bias and to reflect current clinical practice. Refusals to participate were very rare; however, the exact number of women who declined was not systematically recorded, and a precise non-participation rate cannot therefore be reported.

The inclusion criteria were age ≥ 18 years, singleton pregnancy, third trimester of pregnancy (28–39 weeks of gestation), the capacity to understand and complete the assessment instruments, and the absence of cognitive or language difficulties that might have affected the validity of responses.

The exclusion criteria were severe obstetric complications, a self-reported history of severe mental disorders (for example, psychotic disorders or bipolar disorder), current psychotropic treatment, and severe chronic medical conditions that might have substantially influenced psychological functioning or participation in the study. Obstetric eligibility criteria were verified by the attending obstetricians, while psychological eligibility criteria were verified by the research team on the basis of the available medical information and participants’ self-reports, in accordance with the study protocol.

### 2.3. Instruments and Psychosocial Variables Assessed

The variables examined in the present study were assessed using two standardised psychometric instruments and an original anthropological questionnaire. In line with the study objectives, the analyses included both variables derived from validated psychometric instruments (the Adult Attachment Scale and the Edinburgh Postnatal Depression Scale) and study-specific psychosocial variables operationalised on the basis of the anthropological questionnaire. All instruments were administered to participants within a single assessment session.

#### 2.3.1. The Anthropological Questionnaire

The anthropological questionnaire was an original instrument developed during the first stage of the research project in order to characterise the psychosocial, relational and obstetric context of pregnancy. It was designed on the basis of the literature on perinatal mental health, attachment theory and medical anthropology, and its content was reviewed by an interdisciplinary panel comprising clinical psychologists, anthropologists and physicians to ensure content validity. Prior to its use in the main study, the questionnaire was piloted with a group of 10 pregnant women, and the resulting observations were incorporated into the final version.

The final version comprised 50 items organised into domains covering socio-demographic characteristics, obstetric history, family and relational context, lifestyle, sleep quality, pregnancy planning, perceived partner support, emotional history, and representations of motherhood and of the antenatal relationship with the child.

Five psychosocial variables derived from the questionnaire were analysed in the present study, selected on the basis of their relevance to the research hypotheses: pregnancy planning, perceived partner support, perception of motherhood, previous emotional history and sleep problems. Each variable was operationalised through a single item with predefined response options, following a coding scheme established in the research protocol:

- Pregnancy planning was assessed through a dichotomous item (planned/unplanned pregnancy).
- Perceived partner support was assessed through a dichotomous item concerning the presence or absence of emotional support from the partner during pregnancy (present/absent).
- Perception of motherhood was assessed through an item with three response options reflecting the subjective attitude towards pregnancy and the maternal role: positive (“I want this child, I am happy”), ambivalent (“I have some worries or fears”) and negative (“I regret being pregnant”).
- Previous emotional history was assessed through a dichotomous item concerning the presence or absence of emotional difficulties prior to pregnancy (present/absent).
- Sleep problems during pregnancy were assessed through a dichotomous item concerning the presence or absence of sleep difficulties experienced during pregnancy (present/absent).

These variables were measured using single items not derived from validated psychometric scales; this operationalisation is acknowledged as a limitation of the study (see Section 4.8).

#### 2.3.2. Edinburgh Postnatal Depression Scale (EPDS)

Antenatal depressive symptoms were assessed using the Edinburgh Postnatal Depression Scale (EPDS), a 10-item screening instrument developed by Cox et al. [29]. For the Romanian population, the version adapted and validated for the antenatal period (EPDS-R) was used [30]. Each item is rated on a four-point Likert scale, with total scores ranging from 0 to 30; higher scores indicate greater severity of depressive symptoms. The EPDS is a screening instrument and does not establish a clinical diagnosis of depression. A score of ≥14 was taken to indicate a positive screen and defined the dependent variable in the regression analyses [30]. Item 10 (self-harm ideation) was monitored separately, with any non-zero response prompting referral for clinical assessment. In the present sample, no participant reported self-harm ideation on this item. Internal consistency in the present sample was good (Cronbach’s α = 0.85).

#### 2.3.3. Adult Attachment Scale (AAS)

Participants’ attachment style was assessed using the Adult Attachment Scale (AAS), developed by Collins and Read [31]. The instrument comprises 18 items organised into three subscales corresponding to the dimensions of adult attachment—Close, Depend and Anxiety—rated on a five-point Likert scale ranging from 1 (“strongly disagree”) to 5 (“strongly agree”). Mean scores were calculated for each of the three subscales for every participant, and the predominant attachment style was defined as the subscale with the highest mean score, according to a pre-specified decision rule. The resulting classification (secure, avoidant or anxious–ambivalent) was used as a categorical variable in the inferential analyses. Internal consistency of the AAS in the present sample was acceptable (Cronbach’s α = 0.73).

### 2.4. Data Collection Procedure

After written informed consent had been obtained, participants completed all study instruments within a single assessment session. All instruments were completed individually, in a setting that ensured confidentiality of responses and standardised administration conditions. Questionnaires were completed either in printed form or through a secure electronic platform, according to participants’ preference and availability. Administration of the instruments was standardised and coordinated by a clinical psychologist. The mean completion time for all instruments was approximately 30–35 min.

Following data collection, all questionnaires were checked for completeness, coded and entered into the database used for the statistical analyses. All data were anonymised prior to statistical processing, with each participant being identified solely by a unique numerical code.

### 2.5. Ethical Considerations

The study was conducted in accordance with the Declaration of Helsinki and with the ethical principles applicable to research involving human participants. The study protocol was approved by the Ethics Committee of the “Francisc I. Rainer” Institute of Anthropology, Romanian Academy (Approval No. 281/2021 of 12 October 2021), before recruitment commenced.

Participation was voluntary. Before enrolment, all participants received information regarding the objectives and procedures of the research, the voluntary nature of participation, the right to withdraw at any time without consequences for their medical care, and the measures adopted to protect confidentiality and personal data. Written informed consent was obtained before any research procedure was initiated.

All data were anonymised prior to statistical processing and used exclusively for the purposes of the present research, in accordance with the principles of confidentiality and personal data protection.

### 2.6. Statistical Analysis

Statistical analyses were performed using IBM SPSS Statistics, version 29.0 (IBM Corp., Armonk, NY, USA) for descriptive statistics and bivariate analyses, while penalized logistic regression was carried out in R, version 4.6.1 (R Foundation for Statistical Computing, Vienna, Austria), using the logistf package (version 1.26.1).

Continuous variables were presented as mean ± standard deviation (SD) or as median and interquartile range (IQR), depending on data distribution, and categorical variables were expressed as frequencies and percentages. The distribution of continuous variables was assessed by graphical inspection and the Shapiro–Wilk test. There were no missing data: all 140 participants provided complete responses for every variable analysed, and no imputation or case exclusion was required.

Associations between categorical variables were examined using Pearson’s χ^2^ test, and effect size was estimated using Cramér’s V coefficient, for which 95% confidence intervals were obtained using the noncentral chi-square method. In contingency tables in which more than 20% of cells had expected frequencies below 5, or in which cells with zero observed frequency were present, the significance of the association was additionally verified using the Fisher–Freeman–Halton exact test (Monte Carlo estimation, 10,000 samples), in order to ensure the validity of inference under sparse-frequency conditions. Relationships between ordinal variables, or between continuous variables not following a normal distribution, were examined using Spearman’s correlation coefficient.

Factors independently associated with a positive antenatal depression screen were examined using penalized logistic regression with Firth’s method, with the presence of an EPDS score ≥ 14 as the dependent variable. This method was chosen after quasi-complete separation was identified in the initial maximum likelihood logistic regression models, a phenomenon reflected in unstable coefficient estimates and extreme odds ratio values. Firth penalized logistic regression reduces the bias of maximum likelihood estimates and yields finite, stable estimates in situations characterised by data separation or a small number of events.

Attachment style was not entered as a predictor in the multivariable model: the absence of any case with EPDS ≥ 14 in the secure and avoidant categories produced complete separation, which is incompatible with stable coefficient estimation. The relationship between attachment style and depressive symptoms was therefore analysed at the bivariate level only.

Construction of the multivariable model followed a parsimonious approach, guided by the study hypotheses, the clinical relevance of the variables and the results of the bivariate analyses. The number of available events (21 participants with EPDS ≥ 14) limited the number of predictors that could be estimated reliably; in line with recommendations on the number of events per variable, the main model included two predictors (perception of motherhood and previous emotional history). Collinearity between the two predictors was assessed prior to estimation: although they were moderately associated (φ = 0.537), the variance inflation factor was 1.41 (tolerance = 0.711), well below conventional thresholds, indicating no problematic collinearity. Additional models incorporating pregnancy planning, perceived partner support and sleep problems were evaluated as sensitivity analyses; as the inclusion of these variables did not alter the direction, significance or magnitude of the principal association, the most parsimonious model was retained.

Regression results are presented as odds ratios (OR) with 95% confidence intervals (95% CI) and *p* values. Overall model significance was assessed using the likelihood ratio test. All statistical tests were two-tailed, and the threshold for statistical significance was set at *p* < 0.05.

Given the cross-sectional design of the study, all results were interpreted as statistical associations between variables, without permitting causal inference.

## 3. Results

### 3.1. Characteristics of the Study Sample

The sample comprised 140 pregnant women in the third trimester of pregnancy, with a mean age of 33.56 ± 3.19 years (range 28–40 years; median 34.0). Most participants had university or postgraduate education (73.6%), lived in an urban setting (69.3% in Bucharest) and were married (74.3%) or in a stable relationship (22.9%). With regard to obstetric characteristics, 60.0% had one child, 37.1% were primiparous and 2.9% had two children; approximately half of the pregnancies had been planned (48.6%). Most women reported the presence of partner support (72.9%).

With regard to the psychosocial variables, perception of motherhood was predominantly ambivalent (58.6%), followed by negative (21.4%) and positive (20.0%), while 48.6% of participants reported a history of emotional difficulties prior to pregnancy. The socio-demographic, obstetric and psychosocial characteristics of the sample are presented in Table 1.

### 3.2. Distribution of Attachment Styles and Depressive Symptoms

**Figure 1:**
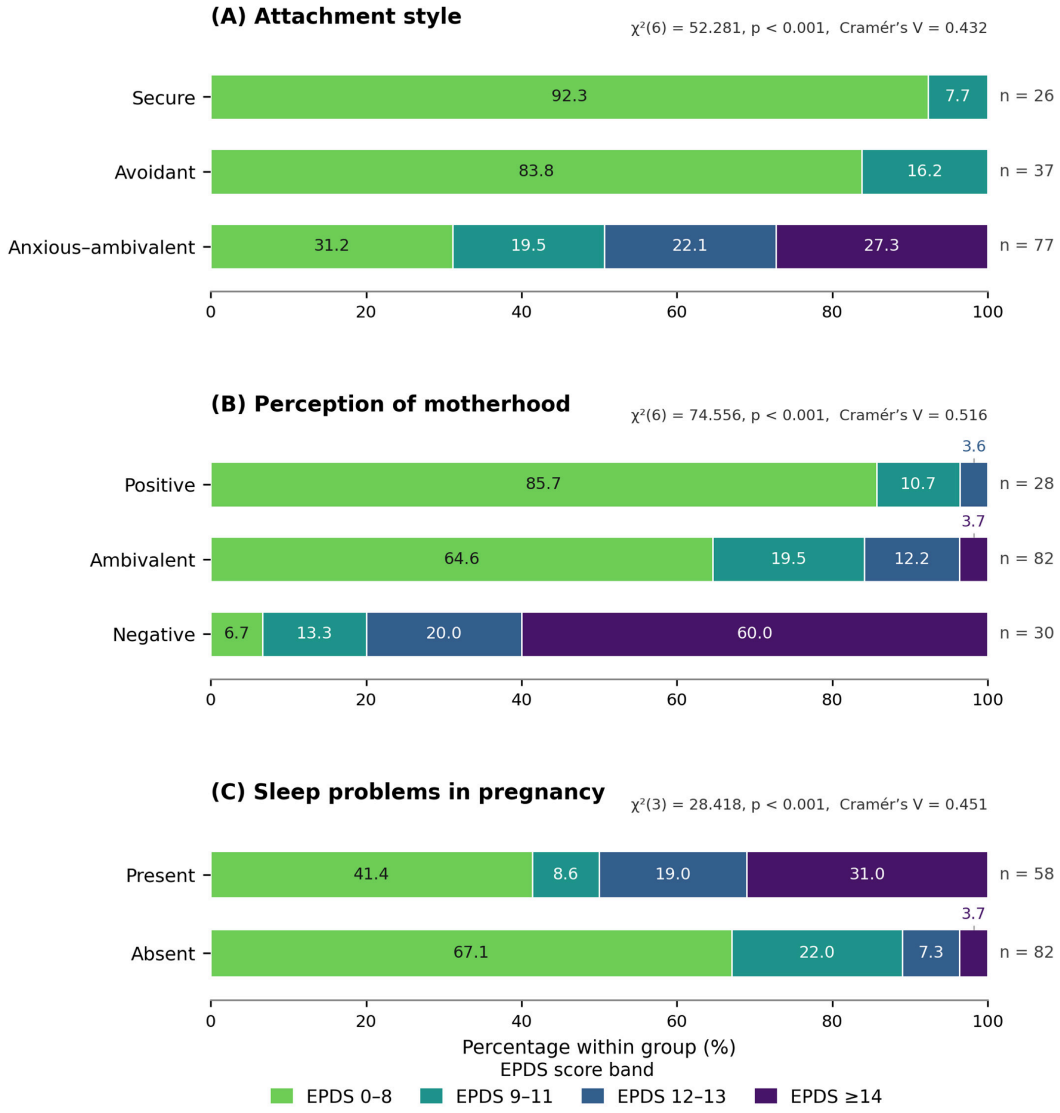
Distribution of Edinburgh Postnatal Depression Scale (EPDS) score bands within categories of (**A**) attachment style, (**B**) perception of motherhood, and (**C**) sleep problems during pregnancy (*N* = 140). Percentages are calculated within each group and may not sum to 100% owing to rounding. Every non-zero category is labelled; labels for very small segments are placed above the bar. Chi-square statistics and Cramér’s V are shown for each association. EPDS, Edinburgh Postnatal Depression Scale.

Within the sample, the anxious–ambivalent attachment style was the most frequent, identified in 77 participants (55.0%), followed by the avoidant style (37 participants; 26.4%) and the secure style (26 participants; 18.6%). As assessed by the Edinburgh Postnatal Depression Scale (EPDS), antenatal depressive symptoms were absent in slightly more than half of the participants (56.4% with scores of 0–8), whereas 21 participants (15.0%) obtained EPDS scores of ≥14, corresponding to a positive antenatal depression screen. The distribution of EPDS scores across attachment style, perception of motherhood and sleep problems is summarised in Figure 1A–C.

The association analysis revealed a significant relationship between attachment style and the level of antenatal depressive symptoms (χ^2^ = 52.281; df = 6; *p* < 0.001; Cramér’s V = 0.432). As more than 20% of cells had expected frequencies below 5, significance was confirmed using the Fisher–Freeman–Halton exact test (*p* < 0.001). No participant with a secure attachment style obtained an EPDS score of ≥14, and elevated scores occurred exclusively among women with an anxious–ambivalent style, of whom 27.3% had a positive screen (EPDS ≥ 14). The full distribution of EPDS categories by attachment style (Figure 1A) is presented in Table 2.

### 3.3. Associations Between Psychosocial Factors and Antenatal Depressive Symptoms

The bivariate analyses were extended to examine the associations between antenatal depressive symptoms and the principal psychosocial variables assessed: perception of motherhood and a history of emotional difficulties.

Perception of motherhood (Figure 1B) was significantly associated with the severity of depressive symptoms (χ^2^ = 74.556; df = 6; *p* < 0.001; Cramér’s V = 0.516; confirmed by the Fisher–Freeman–Halton exact test, *p* < 0.001). Participants with a positive perception predominantly obtained low EPDS scores (85.7% in the 0–8 range), with no case of a positive screen. By contrast, among women with a negative perception, 60.0% obtained EPDS scores of ≥14. The full distribution is presented in Table 3.

A significant association was also observed between a history of emotional difficulties and the level of depressive symptoms (χ^2^ = 26.351; df = 3; *p* < 0.001; Cramér’s V = 0.434). Among participants with a previous emotional history, 29.4% obtained EPDS scores of ≥14, compared with only 1.4% of women without such a history. The full distribution is presented in Table 4.

These findings indicated that attachment style, perception of motherhood and previous emotional history were all associated with the severity of antenatal depressive symptoms. Variables of statistical and clinical relevance were subsequently entered into the multivariable analysis in order to assess their independent association with a positive antenatal depression screen (EPDS ≥ 14).

### 3.4. Sleep Problems and Antenatal Depressive Symptoms

Sleep problems reported during pregnancy (Figure 1C) were significantly associated with the severity of antenatal depressive symptoms (χ^2^ = 28.418; df = 3; *p* < 0.001; Cramér’s V = 0.451). Among participants reporting sleep disturbances, 31.0% had a positive screen (EPDS ≥ 14), compared with only 3.7% of those without such difficulties. The full distribution is presented in Table 5.

Sleep problems were also differentially distributed across attachment styles: they were reported by 54.5% of participants with anxious–ambivalent attachment and 37.8% of those with avoidant attachment, but by only 7.7% of those with secure attachment. This pattern suggests that sleep difficulties extend across both insecure attachment styles, and not solely the anxious–ambivalent style.

### 3.5. Factors Independently Associated with a Positive Antenatal Depression Screen

To identify the factors independently associated with a positive antenatal depression screen (EPDS ≥ 14), a Firth penalized logistic regression model was constructed in accordance with the statistical analysis plan. Attachment style could not be entered as a predictor because of complete separation (no case with EPDS ≥ 14 in the secure and avoidant categories); its relationship with depressive symptoms was therefore analysed at the bivariate level only (Table 2). The multivariable model included perception of motherhood and a history of emotional difficulties.

**Figure 2:**
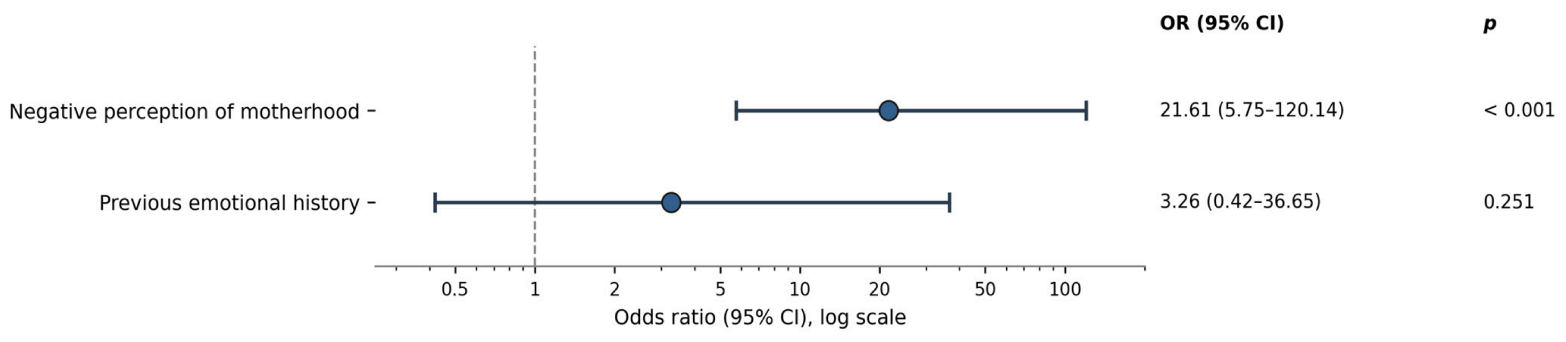
Forest plot of the Firth penalized logistic regression model for screen-positive antenatal depression (EPDS ≥ 14). Points represent odds ratios (OR); horizontal lines represent 95% confidence intervals on a logarithmic scale. The dashed vertical line indicates OR = 1 (no association). CI, confidence interval; EPDS, Edinburgh Postnatal Depression Scale; OR, odds ratio.

The overall model was statistically significant (likelihood ratio χ^2^ = 49.47; df = 2; *p* < 0.001; *n* = 140). Negative perception of motherhood remained independently associated with a positive screen for antenatal depressive symptoms (OR = 21.61; 95% CI, 5.75–120.14; *p* < 0.001). A history of emotional difficulties did not remain independently associated after adjustment (OR = 3.26; 95% CI, 0.42–36.65; *p* = 0.251). The Firth model estimates, together with their 95% confidence intervals, are displayed graphically in Figure 2.

The width of the confidence interval for perception of motherhood reflects the small number of events and indicates an estimate that is robust in direction and significance but imprecise in magnitude, requiring confirmation in larger samples.

Sensitivity analyses were conducted by entering additional variables into the model. Sleep problems, which showed a strong bivariate association with a positive screen (Section 3.4), did not remain independently associated after adjustment for perception of motherhood and previous emotional history (OR = 2.63; 95% CI, 0.58–13.47; *p* = 0.209), and the likelihood ratio test indicated no significant improvement in model fit (LR χ^2^ = 2.11; df = 1; *p* = 0.146). Models incorporating pregnancy planning and perceived partner support likewise did not alter the direction, significance or magnitude of the principal association. Accordingly, although sleep problems represent a robust bivariate marker of depressive vulnerability, their informative contribution overlaps with that of the other psychosocial dimensions assessed, and the most parsimonious model was retained.

The full results are presented in Table 6.

## 4. Discussion

### 4.1. Main Findings

The present study examined psychosocial factors associated with antenatal depressive symptoms in a sample of women in the third trimester of pregnancy, highlighting the role of perception of motherhood, previous emotional history, attachment style and sleep problems in antenatal psychological vulnerability. The importance of identifying these factors early is supported by evidence documenting the impact of perinatal mental health disorders on children’s emotional, cognitive and behavioural development [4,32], as well as the association of maternal depression with unfavourable perinatal outcomes [33]. The high prevalence of perinatal depressive symptoms, documented in syntheses at a global level [34,35,36], underlines the public health dimension of this problem, and its determinants vary according to national socio-economic context [37].

The bivariate analyses revealed consistent associations between these variables and the severity of depressive symptoms, in keeping with the contemporary conceptualisation of antenatal depression as a multifactorial condition situated at the intersection of cognitive, affective and relational factors [5]. Specifically, anxious–ambivalent attachment and negative perceptions of motherhood were associated with the highest levels of depressive symptoms, whereas secure attachment and positive maternal representations were associated with more adaptive emotional profiles.

In the multivariable analysis using Firth penalized logistic regression, negative perception of motherhood remained independently associated with a positive screen for antenatal depressive symptoms (EPDS ≥ 14). This finding suggests that the cognitive and emotional representations of motherhood may reflect deeper mechanisms of affective and relational organisation involved in antenatal psychological adaptation [17,18].

By simultaneously integrating several psychological and relational dimensions, the study supports the value of a multidimensional approach to perinatal mental health and the relevance of an integrated assessment of emotional vulnerability during the antenatal period.

### 4.2. Attachment and Antenatal Depressive Symptoms

One of the most relevant findings is the strong association between attachment style and the severity of antenatal depressive symptoms. Participants with anxious–ambivalent attachment presented the highest levels of depressive symptoms, whereas women with secure attachment did not reach thresholds indicative of a positive screen.

These findings accord with the literature showing that attachment insecurity is associated with difficulties in emotional regulation, interpersonal hypersensitivity and heightened vulnerability to psychological stress [11,38]. During pregnancy, the activation of attachment systems and the relational reorganisation specific to the transition to motherhood may intensify the need for emotional security and interpersonal support, thereby increasing the susceptibility of women with insecure attachment to depressive and anxiety symptoms [13,39]. Recent studies suggest that emotional regulation may mediate the relationship between attachment insecurity and depressive symptoms during pregnancy [40,41].

From a theoretical standpoint, anxious–ambivalent attachment is characterised by hyperactivation of attachment strategies, excessive preoccupation with close relationships and a heightened need for external validation [11]. Against the background of the biological and emotional changes specific to pregnancy, these characteristics may be associated with increased emotional distress. Secure attachment, by contrast, is associated with adaptive coping strategies, more effective affect regulation and appropriate use of social support [15,41], as well as with more favourable health behaviours during pregnancy [42].

It should be emphasised that, owing to complete separation of the data—no case with EPDS ≥ 14 in the secure and avoidant groups—attachment style could not be included in the multivariable model, and its relationship with depressive symptoms was analysed at the bivariate level only. Consequently, although the observed association is strong, it is interpreted as a statistical relationship, without implying a causal direction.

Similar findings have recently been reported in a Romanian sample of pregnant women, in which anxious–ambivalent attachment was associated with elevated levels of antenatal depressive symptoms in the third trimester [43]. Comparable patterns have also been described in different socio-cultural contexts, including a perinatal cohort in Japan, where attachment insecurity was associated with a positive EPDS screen [44], suggesting that attachment-related vulnerability may constitute a cross-culturally relevant factor.

### 4.3. Perception of Motherhood as a Central Dimension of Emotional Adaptation

Another important finding is the strong association between perception of motherhood and the severity of antenatal depressive symptoms—and, in the Firth model, the only variable that remained independently associated with a positive screen. Participants reporting negative representations of motherhood presented the highest levels of depressive symptoms, whereas positive perceptions were associated with more adaptive emotional profiles.

These findings suggest that the way in which women internalise the experience of pregnancy and anticipate the transition to the maternal role may be associated with both emotional adaptation and psychological vulnerability. Negative maternal representations may reflect emotional insecurity, anticipatory anxiety and difficulties in integrating maternal identity [16,45].

Convergent findings have recently been reported in studies of similar populations, which identified associations between negative representations of motherhood, attachment insecurity and antenatal depressive symptoms [27]. In the present sample, participants with secure attachment predominantly reported positive perceptions of motherhood, whereas anxious–ambivalent attachment was associated almost exclusively with negative or ambivalent representations. These findings are consistent with theoretical models describing motherhood as a complex process of psychological and relational reorganisation [18], a process also reflected at the level of the neurobiological changes associated with pregnancy [46]. The quality of antenatal maternal representations has, in turn, been associated with the mother–infant bond in the postnatal period [47].

The reorganisation of family functioning and of the support network during the transition to parenthood has been described in the Romanian literature through the circumplex model of marital and family systems [26], which offers a framework for understanding how family cohesion and adaptability relate to the emotional resources available during the perinatal period.

### 4.4. The Role of Previous Emotional History

Previous emotional history was significantly associated with antenatal depressive symptoms at the bivariate level: among participants with a previous emotional history, 29.4% had a positive screen (EPDS ≥ 14), compared with only 1.4% of those without such a history (χ^2^ = 26.351; *p* < 0.001; Cramér’s V = 0.434). Moreover, of the 21 participants with EPDS ≥ 14, 20 (95.2%) had reported a previous emotional history, underlining the clinical relevance of this dimension as an indicator of vulnerability.

In the adjusted multivariable model, however, previous emotional history did not remain independently associated with a positive screen (OR = 3.26; 95% CI, 0.42–36.65; *p* = 0.251). This pattern is explained by the substantial overlap between previous emotional history and the other psychosocial dimensions: none of the participants with secure attachment reported a previous emotional history, whereas 63.6% of those with anxious–ambivalent attachment did so. The association of previous emotional history therefore appears to overlap with attachment organisation and perception of motherhood, rather than acting independently. From a clinical perspective, this suggests that previous emotional history remains a useful signal of vulnerability in antenatal assessment, although its informative value partly overlaps with that of attachment style—an aspect warranting confirmation in larger samples [19,48]. The literature further suggests that early adverse experiences may contribute to maternal emotional vulnerability during the perinatal period [49].

### 4.5. Contextual and Relational Factors

With regard to contextual factors, pregnancy planning and perceived partner support were evaluated as additional variables in sensitivity analyses. Their inclusion in the multivariable model did not alter the direction, significance or magnitude of the principal association, and neither reached the threshold of statistical significance after adjustment.

This finding should be interpreted with caution. The relationship between social support and perinatal mental health is complex and probably mediated by additional factors, including relationship quality, individual coping strategies and participants’ interpersonal histories. Recent meta-analyses have shown that low social support is associated with perinatal depression and anxiety, although the magnitude of this effect may vary according to population characteristics and socio-cultural context [50,51]. The quality of the couple relationship and personality traits may, in turn, modulate this association [52]. The absence of an independent association in the present sample may also reflect the small size of the subgroups and the measurement of support through a single item.

Sleep problems reported during pregnancy were likewise strongly associated with antenatal depressive symptoms at the bivariate level, but did not remain independently associated after adjustment for perception of motherhood and previous emotional history. This pattern is consistent with the literature describing a bidirectional and closely intertwined relationship between sleep disturbances and perinatal depressive symptoms [24]. The differential distribution of sleep problems across attachment styles—more frequent in anxious–ambivalent and avoidant attachment—suggests that sleep difficulties may reflect a shared substrate of emotional vulnerability rather than an independent correlate. From a clinical perspective, the presence of sleep disturbances may serve as an accessible warning signal in antenatal assessment, even though its informative value overlaps with that of other psychosocial dimensions. The frequent comorbidity of anxiety and depression during the perinatal period may also contribute to the overlap between these dimensions [53].

### 4.6. Integration of Findings and Contribution of the Study

The findings support an integrated model of antenatal depressive symptoms, in which psychological, cognitive and relational variables are convergently associated with emotional vulnerability during pregnancy. Perception of motherhood, previous emotional history, attachment style and sleep problems do not appear to operate independently, but rather to delineate a complex psychological profile associated with antenatal depressive symptoms.

Investigating these relationships in a sample from an Eastern European context contributes to reducing the existing gap in the regional literature on antenatal mental health. The study forms part of an interdisciplinary line of research developed in Romania, at the intersection of medical anthropology, clinical psychology and perinatal health [43].

### 4.7. Clinical Implications

From a clinical perspective, the findings support the value of implementing systematic psychological screening during the antenatal period, encompassing not only the assessment of depressive symptoms but also the exploration of perception of motherhood, attachment style, emotional history and sleep quality. It should be emphasised that the EPDS is a screening instrument rather than a diagnostic one; a score of ≥14 indicates the need for further clinical assessment, not a diagnosis of depression [54].

Integrating these dimensions may facilitate more accurate identification of women at increased psychological risk and the development of personalised preventive interventions. Current international guidelines recommend the inclusion of depression and anxiety screening in routine antenatal assessment, particularly for women with psychological risk factors or a relevant emotional history [7,55]. Present evidence also supports offering preventive psychological interventions to women at increased risk [56,57], although effective access to perinatal mental health services remains limited in routine practice [58]. These clinical implications should be regarded as preliminary: given the cross-sectional design and the small number of positive screens, the present findings require confirmation in larger, longitudinal and multicentre studies before they can be translated into specific clinical recommendations or implemented in routine antenatal care.

### 4.8. Limitations

The study has several limitations. First, the cross-sectional design does not permit causal relationships between variables to be established, only the identification of statistical associations. Second, the use of self-report instruments may introduce response biases, including social desirability. Furthermore, although the EPDS is a well-validated screening instrument, it does not establish a clinical diagnosis of depression and may yield false-positive or false-negative classifications depending on the selected cut-off and the clinical context; the outcome analysed here therefore reflects a positive screen rather than diagnosed depression, and some outcome misclassification cannot be excluded. In addition, the assessment of certain psychosocial variables (perception of motherhood, partner support, previous emotional history, pregnancy planning and sleep problems) through single, non-validated items limits the depth and reliability of measurement.

The attachment scale (AAS) does not have a dedicated validation in the Romanian population of mothers; internal consistency in the present sample was nonetheless acceptable (α = 0.73). The sample, predominantly urban and relatively homogeneous in socio-cultural terms, limits the generalisability of the findings to women from rural settings or other socio-economic backgrounds. Vulnerable populations, such as migrant or refugee women, present distinct risk profiles and remain insufficiently represented in the present study [59,60].

Certain potentially relevant variables—including socio-economic status, employment, relationship satisfaction, previous obstetric complications and psychiatric treatment history—were not assessed at this stage of the project. Residual confounding therefore cannot be excluded.

The small number of events (21 cases with EPDS ≥ 14) limited the number of predictors that could be estimated and resulted in wide confidence intervals; although Firth’s method yielded finite and stable estimates under conditions of separation, the magnitude of the odds ratios should be interpreted with caution and requires confirmation in larger samples. Complete separation in the attachment × EPDS cells necessitated bivariate analysis of this relationship.

### 4.9. Future Research Directions

Future studies should employ longitudinal designs, allowing investigation of the course of depressive symptoms throughout pregnancy and the postpartum period, as well as examination of the directionality of the relationships between the psychological variables examined [61]. The integration of biomarkers (neuroendocrine, inflammatory and physiological indicators of stress) may contribute to a better understanding of the mechanisms involved in perinatal emotional vulnerability. Investigating dimensions specific to the third trimester, such as fear of childbirth, could refine explanatory models of emotional vulnerability in the period preceding childbirth [62]. Extending research to more socio-demographically diverse populations and using validated multidimensional instruments for perception of motherhood and relational support would strengthen the validity of the conclusions.

## 5. Conclusions

The present study examined the psychosocial factors associated with a positive antenatal depression screen in a sample of women in the third trimester of pregnancy, integrating attachment style, perception of motherhood, previous emotional history and sleep problems. The findings indicate that negative perception of motherhood was the only factor that remained independently associated with a positive antenatal depression screen (EPDS ≥ 14). At the bivariate level, anxious–ambivalent attachment, previous emotional history and sleep disturbances were also significantly associated, reflecting a shared substrate of emotional vulnerability. From a methodological standpoint, the use of Firth penalized logistic regression allowed stable estimates to be obtained under conditions of data separation and a small number of events.

From a clinical perspective, these findings support the value of an integrated psychosocial assessment during the antenatal period, complementing standard EPDS screening with the exploration of perception of motherhood, attachment style, emotional history and sleep quality. Early identification of women with a profile of psychological vulnerability may facilitate timely referral to perinatal mental health services and the development of personalised preventive interventions. Future studies with longitudinal designs and larger, more diverse samples are needed to confirm these associations and to investigate the directionality of the relationships identified.

## Figures and Tables

**Table 1:** Sociodemographic, obstetric, and psychosocial characteristics of the study sample (*N* = 140).

Characteristic	Category	*n* (%)
Age (years)	Mean ± SD	33.56 ± 3.19
	Range	28–40
Age group (years)	28–32	54 (38.6)
	33–37	71 (50.7)
	38–40	15 (10.7)
Education	Secondary (high school)	25 (17.9)
	University/postgraduate	103 (73.6)
	Doctoral	12 (8.6)
Residence	Bucharest (capital)	97 (69.3)
	Other cities	43 (30.7)
Marital status	Married	104 (74.3)
	Stable relationship	32 (22.9)
	Single mother	4 (2.9)
Number of children	0 (primiparous)	52 (37.1)
	1	84 (60.0)
	2	4 (2.9)
Gestational stage	Early third trimester (28–32 wk)	86 (61.4)
	Late third trimester (33–39 wk)	54 (38.6)
Pregnancy planning	Planned	68 (48.6)
	Unplanned	72 (51.4)
Partner support	Present	102 (72.9)
	Absent	38 (27.1)
Perception of motherhood	Positive	28 (20.0)
	Ambivalent	82 (58.6)
	Negative	30 (21.4)
Previous emotional history	Present	68 (48.6)
	Absent	72 (51.4)
Attachment style (AAS)	Secure	26 (18.6)
	Avoidant	37 (26.4)
	Anxious–ambivalent	77 (55.0)
Depressive symptoms (EPDS)	0–8 (none)	79 (56.4)
	9–11 (possible)	23 (16.4)
	12–13 (high probability)	17 (12.1)
	≥14 (positive screen)	21 (15.0)

Percentages may not sum to 100% owing to rounding. Abbreviations: AAS, Adult Attachment Scale; EPDS, Edinburgh Postnatal Depression Scale; SD, standard deviation; wk, weeks.

**Table 2:** Distribution of EPDS categories by attachment style (*N* = 140).

Attachment Style	0–8 *n* (%)	9–11 *n* (%)	12–13 *n* (%)	≥14 *n* (%)	Total *n* (%)
Secure	24 (92.3)	2 (7.7)	0 (0.0)	0 (0.0)	26 (100)
Avoidant	31 (83.8)	6 (16.2)	0 (0.0)	0 (0.0)	37 (100)
Anxious–ambivalent	24 (31.2)	15 (19.5)	17 (22.1)	21 (27.3)	77 (100)
**Total**	**79 (56.4)**	**23 (16.4)**	**17 (12.1)**	**21 (15.0)**	**140 (100)**

χ^2^ = 52.281, df = 6, *p* < 0.001; Cramér’s V = 0.432 (95% CI, 0.290–0.531); Fisher–Freeman–Halton exact test *p* < 0.001. Row percentages may not sum to 100% owing to rounding. Confidence intervals for Cramér’s V were obtained using the noncentral chi-square method. Bold indicates the total row for the whole sample (*N* = 140). Abbreviations: CI, confidence interval; EPDS, Edinburgh Postnatal Depression Scale; df, degrees of freedom.

**Table 3:** Distribution of EPDS categories by perception of motherhood (*N* = 140).

Perception of Motherhood	0–8 *n* (%)	9–11 *n* (%)	12–13 *n* (%)	≥14 *n* (%)	Total *n* (%)
Positive	24 (85.7)	3 (10.7)	1 (3.6)	0 (0.0)	28 (100)
Ambivalent	53 (64.6)	16 (19.5)	10 (12.2)	3 (3.7)	82 (100)
Negative	2 (6.7)	4 (13.3)	6 (20.0)	18 (60.0)	30 (100)
**Total**	**79 (56.4)**	**23 (16.4)**	**17 (12.1)**	**21 (15.0)**	**140 (100)**

χ^2^ = 74.556, df = 6, *p* < 0.001; Cramér’s V = 0.516 (95% CI, 0.379–0.617); Fisher–Freeman–Halton exact test *p* < 0.001. Row percentages may not sum to 100% owing to rounding. Confidence intervals for Cramér’s V were obtained using the noncentral chi-square method. Bold indicates the total row for the whole sample (*N* = 140). Abbreviations: CI, confidence interval; EPDS, Edinburgh Postnatal Depression Scale; df, degrees of freedom.

**Table 4:** Distribution of EPDS categories by previous emotional history (*N* = 140).

Previous Emotional History	0–8 *n* (%)	9–11 *n* (%)	12–13 *n* (%)	≥14 *n* (%)	Total *n* (%)
Present	28 (41.2)	9 (13.2)	11 (16.2)	20 (29.4)	68 (100)
Absent	51 (70.8)	14 (19.4)	6 (8.3)	1 (1.4)	72 (100)
**Total**	**79 (56.4)**	**23 (16.4)**	**17 (12.1)**	**21 (15.0)**	**140 (100)**

χ^2^ = 26.351, df = 3, *p* < 0.001; Cramér’s V = 0.434 (95% CI, 0.246–0.585). Row percentages may not sum to 100% owing to rounding. Confidence intervals for Cramér’s V were obtained using the noncentral chi-square method. Bold indicates the total row for the whole sample (*N* = 140). Abbreviations: CI, confidence interval; EPDS, Edinburgh Postnatal Depression Scale; df, degrees of freedom.

**Table 5:** Distribution of EPDS categories by sleep problems during pregnancy (*N* = 140).

Sleep Problems	0–8 *n* (%)	9–11 *n* (%)	12–13 *n* (%)	≥14 *n* (%)	Total *n* (%)
Present	24 (41.4)	5 (8.6)	11 (19.0)	18 (31.0)	58 (100)
Absent	55 (67.1)	18 (22.0)	6 (7.3)	3 (3.7)	82 (100)
**Total**	**79 (56.4)**	**23 (16.4)**	**17 (12.1)**	**21 (15.0)**	**140 (100)**

χ^2^ = 28.418, df = 3, *p* < 0.001; Cramér’s V = 0.451 (95% CI, 0.264–0.602). Row percentages may not sum to 100% owing to rounding. Confidence intervals for Cramér’s V were obtained using the noncentral chi-square method. Bold indicates the total row for the whole sample (*N* = 140). Abbreviations: CI, confidence interval; EPDS, Edinburgh Postnatal Depression Scale; df, degrees of freedom.

**Table 6:** Firth penalized logistic regression: factors independently associated with a positive antenatal depression screen (EPDS ≥ 14).

Predictor	B	OR	95% CI	*p*
Negative perception of motherhood	3.073	21.61	5.75–120.14	<0.001
Previous emotional history	1.182	3.26	0.42–36.65	0.251

Model: Likelihood ratio χ^2^ = 49.47, df = 2, *p* < 0.001; *n* = 140. Reference categories: non-negative perception of motherhood; no previous emotional history. Attachment style was not entered in the multivariable model because of complete separation (no EPDS ≥ 14 cases in the secure and avoidant groups). Sleep problems were tested as an additional predictor but were not independently associated with EPDS ≥ 14 (OR = 2.63; 95% CI, 0.58–13.47; *p* = 0.209; likelihood ratio test *p* = 0.146). Abbreviations: B, regression coefficient; CI, confidence interval; EPDS, Edinburgh Postnatal Depression Scale; OR, odds ratio; df, degrees of freedom.

## Data Availability

The datasets generated and/or analysed during the current study are not publicly available due to privacy and ethical restrictions but are available from the corresponding author on reasonable request. This manuscript reports analyses from the third-trimester stage of a longitudinal cohort; findings from the same cohort have been reported previously using a different analytical model [27], and a separate manuscript reports results from a subsample involving oxytocin quantification, with distinct objectives, endpoints and statistical models. There is no text reuse between these manuscripts.

## Supplementary Materials

The following supporting information can be downloaded at: https://www.mdpi.com/article/10.3390/diagnostics16152320/s1, File S1: STROBE Statement–checklist of items that should be included in reports of observational studies.

## Abbreviations

The following abbreviations are used in this manuscript:

AAS	Adult Attachment Scale
ACOG	American College of Obstetricians and Gynecologists
CI	Confidence Interval
df	Degrees of Freedom
EPDS	Edinburgh Postnatal Depression Scale
EPDS-R	Edinburgh Postnatal Depression Scale, Romanian version
IQR	Interquartile Range
LR	Likelihood Ratio
OR	Odds Ratio
SD	Standard Deviation
SPSS	Statistical Package for the Social Sciences

## References

[1] Yin X., Sun N., Jiang N., Xu X., Gan Y., Zhang J., Qiu L., Yang C., Shi X., Chang J. (2021). Prevalence and associated factors of antenatal depression: Systematic reviews and meta-analyses. Clin. Psychol. Rev..

[2] Woody C.A., Ferrari A.J., Siskind D.J., Whiteford H.A., Harris M.G. (2017). A systematic review and meta-regression of the prevalence and incidence of perinatal depression. J. Affect. Disord..

[3] Slomian J., Honvo G., Emonts P., Reginster J.-Y., Bruyère O. (2019). Consequences of maternal postpartum depression: A systematic review of maternal and infant outcomes. Womens Health.

[4] Biaggi A., Conroy S., Pawlby S., Pariante C.M. (2016). Identifying the women at risk of antenatal anxiety and depression: A systematic review. J. Affect. Disord..

[5] Howard L.M., Khalifeh H. (2020). Perinatal mental health: A review of progress and challenges. World Psychiatry.

[6] O’Connor E., Senger C.A., Henninger M.L., Coppola E., Gaynes B.N. (2019). Interventions to prevent perinatal depression: Evidence report and systematic review for the US Preventive Services Task Force. JAMA.

[7] American College of Obstetricians and Gynecologists’ Committee on Clinical Practice Guidelines–Obstetrics (2023). Screening and diagnosis of mental health conditions during pregnancy and postpartum: ACOG Clinical Practice Guideline No. 4. Obstet. Gynecol..

[8] Stefana A., Mirabella F., Gigantesco A., Camoni L., Perinatal Mental Health Network (2024). The screening accuracy of the Edinburgh Postnatal Depression Scale (EPDS) to detect perinatal depression with and without the self-harm item in pregnant and postpartum women. J. Psychosom. Obstet. Gynaecol..

[9] Levis B., Negeri Z., Sun Y., Benedetti A., Thombs B.D., DEPRESsion Screening Data (DEPRESSD) EPDS Group (2020). Accuracy of the Edinburgh Postnatal Depression Scale (EPDS) for screening to detect major depression among pregnant and postpartum women: Systematic review and meta-analysis of individual participant data. BMJ.

[10] Bowlby J. (1982). Attachment and Loss: Volume 1. Attachment.

[11] Mikulincer M., Shaver P.R. (2016). Attachment in Adulthood: Structure, Dynamics, and Change.

[12] Trombetta T., Giordano M., Santoniccolo F., Vismara L., Della Vedova A.M., Rollè L. (2021). Pre-natal attachment and parent-to-infant attachment: A systematic review. Front. Psychol..

[13] Bianciardi E., Vito C., Betrò S., De Stefano A., Siracusano A., Niolu C. (2020). The anxious aspects of insecure attachment styles are associated with depression either in pregnancy or in the postpartum period. Ann. Gen. Psychiatry.

[14] Galbally M., Watson S., Lewis A.J., Power J., Buus N., van IJzendoorn M.H. (2023). Maternal attachment state of mind and perinatal emotional wellbeing: Findings from a pregnancy cohort study. J. Affect. Disord..

[15] Dagan O., Facompré C.R., Bernard K. (2018). Adult attachment representations and depressive symptoms: A meta-analysis. J. Affect. Disord..

[16] Rollè L., Giordano M., Santoniccolo F., Trombetta T. (2020). Prenatal attachment and perinatal depression: A systematic review. Int. J. Environ. Res. Public Health.

[17] Sechi C., Prino L.E., Rollé L., Lucarelli L., Vismara L. (2022). Maternal attachment representations during pregnancy, perinatal maternal depression, and parenting stress: Relations to child’s attachment. Int. J. Environ. Res. Public Health.

[18] Athan A.M. (2024). A critical need for the concept of matrescence in perinatal psychiatry. Front. Psychiatry.

[19] Racine N., Devereaux C., Cooke J.E., Eirich R., Zhu J., Madigan S. (2021). Adverse childhood experiences and maternal anxiety and depression: A meta-analysis. BMC Psychiatry.

[20] Yang K., Wu J., Chen X. (2022). Risk factors of perinatal depression in women: A systematic review and meta-analysis. BMC Psychiatry.

[21] Underwood L., Waldie K., D’Souza S., Peterson E.R., Morton S. (2016). A review of longitudinal studies on antenatal and postnatal depression. Arch. Womens Ment. Health.

[22] Hetherington E., McDonald S., Williamson T., Patten S.B., Tough S.C. (2018). Social support and maternal mental health at 4 months and 1 year postpartum: Analysis from the All Our Families cohort. J. Epidemiol. Community Health.

[23] Abajobir A.A., Maravilla J.C., Alati R., Najman J.M. (2016). A systematic review and meta-analysis of the association between unintended pregnancy and perinatal depression. J. Affect. Disord..

[24] Fu T., Wang C., Yan J., Zeng Q., Ma C. (2023). Relationship between antenatal sleep quality and depression in perinatal women: A comprehensive meta-analysis of observational studies. J. Affect. Disord..

[25] Rada C., Tarcea M., Rada C., Faludi C. (2015). Vârsta la debutul vieții sexuale, la căsătorie și la nașterea copiilor: Trecut, prezent și viitor în România. Funcții și Disfuncții ale Familiei Contemporane. O Abordare Socio-Psiho-Medicală.

[26] Rada C., Olson D.H. (2016). Circumplex model of marital and family systems (FACES III) in Romania. Annu. Roum. Anthropol..

[27] Soldan N., Glavce C., Kozma A., Stan C., Petrescu M., Maier R., Turcu S. (2025). Maternal attachment and perception of motherhood in relation to third-trimester antenatal depression: A cross-sectional analysis. J. Med. Life.

[28] von Elm E., Altman D.G., Egger M., Pocock S.J., Gøtzsche P.C., Vandenbroucke J.P. (2007). The Strengthening the Reporting of Observational Studies in Epidemiology (STROBE) statement: Guidelines for reporting observational studies. Lancet.

[29] Cox J.L., Holden J.M., Sagovsky R. (1987). Detection of postnatal depression: Development of the 10-item Edinburgh Postnatal Depression Scale. Br. J. Psychiatry.

[30] Wallis A.B., Fernandez R., Oprescu F.I., Cherecheş R.M., Zlati A., Dungy C.I. (2012). Validation of a Romanian scale to detect antenatal depression. Cent. Eur. J. Med..

[31] Collins N.L., Read S.J. (1990). Adult attachment, working models, and relationship quality in dating couples. J. Pers. Soc. Psychol..

[32] Rogers A., Obst S., Teague S.J., Rossen L., Spry E.A., Macdonald J.A., Sunderland M., Olsson C.A., Youssef G., Hutchinson D. (2020). Association between maternal perinatal depression and anxiety and child and adolescent development: A meta-analysis. JAMA Pediatr..

[33] Grigoriadis S., VonderPorten E.H., Mamisashvili L., Tomlinson G., Dennis C.-L., Koren G., Steiner M., Mousmanis P., Cheung A., Radford K. (2013). The impact of maternal depression during pregnancy on perinatal outcomes: A systematic review and meta-analysis. J. Clin. Psychiatry.

[34] Wang Z., Liu J., Shuai H., Cai Z., Fu X., Liu Y., Xiao X., Zhang W., Krabbendam E., Liu S. (2021). Mapping global prevalence of depression among postpartum women. Transl. Psychiatry.

[35] Al-abri K., Edge D., Armitage C.J. (2023). Prevalence and correlates of perinatal depression. Soc. Psychiatry Psychiatr. Epidemiol..

[36] Shorey S., Chee C.Y.I., Ng E.D., Chan Y.H., Tam W.W.S., Chong Y.S. (2018). Prevalence and incidence of postpartum depression among healthy mothers: A systematic review and meta-analysis. J. Psychiatr. Res..

[37] Hahn-Holbrook J., Cornwell-Hinrichs T., Anaya I. (2018). Economic and health predictors of national postpartum depression prevalence: A systematic review, meta-analysis, and meta-regression of 291 studies from 56 countries. Front. Psychiatry.

[38] Verhelst P., Sels L., Lemmens G.M.D., Verhofstadt L. (2024). The role of emotion regulation in perinatal depression and anxiety: A systematic review. BMC Psychol..

[39] Zhang L., Wang L., Yuan Q., Huang C., Cui S., Zhang K., Zhou X. (2021). The mediating role of prenatal depression in adult attachment and maternal-fetal attachment in primigravida in the third trimester. BMC Pregnancy Childbirth.

[40] Burgio S., Cucinella G., Baglio G., Zaami S., Krysiak R., Kowalcze K., Billone V., Gullo G. (2024). Prenatal attachment, personality, and depression in high-risk pregnancies during pandemic emergencies. Healthcare.

[41] Altamura M., Leccisotti I., De Masi L., Gallone F., Ficarella L., Severo M., Biancofiore S., Denitto F., Ventriglio A., Petito A. (2023). Coping as a mediator between attachment and depressive symptomatology either in pregnancy or in the early postpartum period: A structural equation modelling approach. Brain Sci..

[42] Maulina R., Kuo S.-C., Liu C.-Y., Lu Y.Y., Khuzaiyah S., Caparros-Gonzalez R.A. (2025). Does attachment and prenatal depression affect maternal health-promoting lifestyle during pregnancy? A cross-sectional study. Clin. Epidemiol. Glob. Health.

[43] Șoldan N., Glavce C.S., Kozma A., Petrescu M., Stan C., Maier R., Turcu S. (2025). Attachment patterns and antenatal depressive symptoms in Romanian pregnant women: Implications for spa-based rehabilitation programmers. Balneo PRM Res. J..

[44] Oyamada M., Sato M., Nakao T., Sugimoto M. (2026). Attachment style and perinatal depressive symptoms across the perinatal period in Japan. Children.

[45] García-Romero A., Peñacoba C., Catalá P. (2026). Maternal identity and role balance in pregnancy: Construction and validation of the Maternal Role Integration Questionnaire (MRIQ-P). Behav. Sci..

[46] Barba-Müller E., Craddock S., Carmona S., Hoekzema E. (2019). Brain plasticity in pregnancy and the postpartum period: Links to maternal caregiving and mental health. Arch. Womens Ment. Health.

[47] Lutkiewicz K., Bieleninik Ł., Cieślak M., Bidzan M. (2020). Maternal–infant bonding and its relationships with maternal depressive symptoms, stress and anxiety in the early postpartum period in a Polish sample. Int. J. Environ. Res. Public Health.

[48] Silverman M.E., Reichenberg A., Savitz D.A., Cnattingius S., Lichtenstein P., Hultman C.M., Larsson H., Sandin S. (2017). The risk factors for postpartum depression: A population-based study. Depress. Anxiety.

[49] Racine N., Plamondon A., Madigan S., McDonald S., Tough S. (2018). Maternal adverse childhood experiences and infant development. Pediatrics.

[50] Bedaso A., Adams J., Peng W., Sibbritt D. (2021). The relationship between social support and mental health problems during pregnancy: A systematic review and meta-analysis. Reprod. Health.

[51] Sperati A., Passaquindici I., Persico M.E., Di Matteo C., Fasolo M., Lionetti F., Spinelli M. (2025). Maternal depression during the perinatal period: The role of Sensory Processing Sensitivity and social support and its impact on infants’ negative affect. Front. Psychol..

[52] Terzic T., Rus Prelog P., Oblak A., Makovec M.R. (2026). Exploring the influence of personality and partner attachment on perinatal depression and anxiety. BMC Psychol..

[53] Nakić Radoš S., Tadinac M., Herman R. (2018). Anxiety during pregnancy and postpartum: Course, predictors and comorbidity with postpartum depression. Acta Clin. Croat..

[54] Howard L.M., Ryan E.G., Trevillion K., Anderson F., Bick D., Bye A., Byford S., O’Connor S., Sands P., Demilew J. (2018). Accuracy of the Whooley questions and the Edinburgh Postnatal Depression Scale in identifying depression and other mental disorders in early pregnancy. Br. J. Psychiatry.

[55] National Institute for Health and Care Excellence (2014). Antenatal and Postnatal Mental Health: Clinical Management and Service Guidance.

[56] O’Connor E., Rossom R.C., Henninger M., Groom H.C., Burda B.U. (2016). Primary care screening for and treatment of depression in pregnant and postpartum women: Evidence report and systematic review for the US Preventive Services Task Force. JAMA.

[57] US Preventive Services Task Force (2019). Interventions to prevent perinatal depression: US Preventive Services Task Force recommendation statement. JAMA.

[58] Byatt N., Xiao R.S., Dinh K.H., Waring M.E. (2016). Mental health care use in relation to depressive symptoms among pregnant women in the USA. Arch. Womens Ment. Health.

[59] Fellmeth G., Fazel M., Plugge E. (2017). Migration and perinatal mental health in women from low- and middle-income countries: A systematic review and meta-analysis. BJOG.

[60] Catalá P., Gutiérrez L., Écija C., Peñacoba C. (2025). Maternal attachment and perinatal health in refugee women: A systematic review. Psychiatry Int..

[61] Meltzer-Brody S., Howard L.M., Bergink V., Vigod S., Jones I., Munk-Olsen T., Honikman S., Milgrom J. (2018). Postpartum psychiatric disorders. Nat. Rev. Dis. Primers.

[62] Ibrahim H.A., Alshahrani M.S., Elgzar W.T.I. (2024). Determinants of prenatal childbirth fear during the third trimester among low-risk expectant mothers: A cross-sectional study. Healthcare.

