# Supplementary material for: Psychosocial Factors Associated with a Positive Antenatal Depression Screen: A Cross-Sectional Study of Perception of Motherhood, Attachment Style, Emotional History and Sleep Problems
Source: Diagnostics (Basel). 2026 Jul 24;16(15):2320. doi: 10.3390/diagnostics16152320 (PMC13464020; doi:10.3390/diagnostics16152320)
Supplement: Supplementary file 1 [file diagnostics-16-02320-s001.zip › diagnostics-4358384-supplementary.pdf]

STROBE Statement–checklist of items that should be included in reports of observational studies

Manuscript ID: diagnostics-4358384. Title: Psychosocial Factors Associated with a Positive Antenatal Depression Screen: A Cross-Sectional Study of Perception of Motherhood, Attachment Style, Emotional History and Sleep Problems.

	Item No	Recommendation	Reported in
Title and abstract	1	(a) Indicate the study's design with a commonly used term in the title or the abstract<br>(b) Provide in the abstract an informative and balanced summary of what was done and what was found	(a) The study design is stated in the title ("A Cross-Sectional Study") and in the Abstract (Methods section); see also Section 2.1<br>(b) Abstract (structured: Background/Objectives, Methods, Results, Conclusions)
**INTRODUCTION**			
Background/rationale	2	Explain the scientific background and rationale for the investigation being reported	Section 1, paragraphs 1–7
Objectives	3	State specific objectives, including any prespecified hypotheses	Section 1, final paragraph (EPDS ≥14 specified as the dependent variable; two main research questions stated explicitly – which psychosocial factors are associated with a positive screen, and which remain independently associated in the multivariable model; no formal hypotheses were prespecified)
**METHODS**			
Study design	4	Present key elements of study design early in the paper	Section 2.1, first paragraph (cross-sectional observational study within a sequential mixed-methods project)
Setting	5	Describe the setting, locations, and relevant dates, including periods of recruitment, exposure, follow-up, and data collection	Section 2.1, third paragraph (obstetric and antenatal monitoring services, urban Romania; November 2023 – June 2024)
Participants	6	(a) Give the eligibility criteria, and the sources and methods of selection of participants	Section 2.2 (inclusion and exclusion criteria; consecutive enrolment)
Variables	7	Clearly define all outcomes, exposures, predictors, potential confounders, and effect modifiers. Give diagnostic criteria, if applicable	Section 2.3 (all variables defined; EPDS ≥14 as outcome; exact item wording and classification rules for all psychosocial variables)
Data sources/measurement	8	For each variable of interest, give sources of data and details of methods of assessment (measurement). Describe comparability of assessment methods if there is more than one group	Section 2.3 (EPDS, AAS, anthropological questionnaire; versions, scoring procedures, internal consistency); Section 2.4 (administration procedure)

	Item No	Recommendation	Reported in
Bias	9	Describe any efforts to address potential sources of bias	Section 2.2 (consecutive enrolment to reduce selection bias); Section 2.4 (standardised administration, anonymisation); Section 4.8 (self-report and misclassification bias acknowledged)
Study size	10	Explain how the study size was arrived at	Section 2.2 (consecutive recruitment; no a priori power calculation performed – explicitly acknowledged); Section 4.8
Quantitative variables	11	Explain how quantitative variables were handled in the analyses. If applicable, describe which groupings were chosen and why	Section 2.3 (EPDS score bands; derivation of categorical attachment profiles from dimensional subscale scores); Section 2.6
Statistical methods	12	(a) Describe all statistical methods, including those used to control for confounding<br>(b) Describe any methods used to examine subgroups and interactions<br>(c) Explain how missing data were addressed<br>(d) If applicable, describe analytical methods taking account of sampling strategy<br>(e) Describe any sensitivity analyses	(a) Section 2.6 (χ^2, Cramér's V with 95% CI, Fisher–Freeman–Halton exact test, Firth penalized logistic regression; collinearity assessed)<br>(b) Section 3.4 (distribution of sleep problems across attachment styles)<br>(c) Section 2.6 (no missing data; all 140 participants complete)<br>(d) Not applicable (consecutive enrolment, single setting)<br>(e) Section 2.6 and Section 3.5 (sensitivity analyses adding pregnancy planning, partner support and sleep problems)
**RESULTS**			
Participants	13	(a) Report numbers of individuals at each stage of study<br>(b) Give reasons for non-participation at each stage<br>(c) Consider use of a flow diagram	(a) Section 2.2 and Section 3.1 (final sample N = 140; all participants completed all instruments)<br>(b) Section 2.2 (eligibility criteria; all eligible women who agreed were enrolled consecutively; refusals were very rare, although the exact number of women who declined was not systematically recorded)<br>(c) A flow diagram was not included, as no participants were lost between enrolment and analysis
Descriptive data	14	(a) Give characteristics of study participants and information on exposures and potential confounders<br>(b) Indicate number of participants with missing data for each variable of interest	(a) Section 3.1 and Table 1 (socio-demographic, obstetric and psychosocial characteristics)<br>(b) Section 2.6 (no missing data for any variable)
Outcome data	15	Report numbers of outcome events or summary measures	Section 3.2 and Table 1 (21/140, 15.0% with EPDS ≥14; full distribution of EPDS bands)
Main results	16	(a) Give unadjusted estimates and, if applicable, confounder-adjusted estimates and their precision. Make clear which confounders were adjusted for and why they were included<br>(b) Report category boundaries when continuous variables were categorized	(a) Sections 3.2–3.4 and Tables 2–5 (unadjusted associations with 95% CI for Cramér's V); Section 3.5, Table 6 and Figure 2 (adjusted Firth estimates with 95% CI; rationale for predictor selection in Section 2.6)<br>(b) Section 2.3 (EPDS bands 0–8, 9–11, 12–13, ≥14; threshold justification)<br>(c) Absolute proportions reported for every category in Tables 2–5

	Item No	Recommendation	Reported in
		(c) If relevant, consider translating estimates of relative risk into absolute risk	
Other analyses	17	Report other analyses done – e.g. analyses of subgroups and interactions, and sensitivity analyses	Section 3.4 (sleep problems entered as an additional predictor; likelihood ratio test); Section 3.5 (sensitivity analyses with pregnancy planning and partner support)
**DISCUSSION**			
Key results	18	Summarise key results with reference to study objectives	Section 4.1
Limitations	19	Discuss limitations of the study, taking into account sources of potential bias or imprecision. Discuss both direction and magnitude of any potential bias	Section 4.8 (cross-sectional design; self-report and social desirability bias; the EPDS as a screening rather than diagnostic instrument, with potential false-positive/false-negative outcome misclassification; single-item non-validated measures; absence of a priori power calculation; small number of events and wide confidence intervals; complete separation precluding inclusion of attachment style; unmeasured confounders and residual confounding)
Interpretation	20	Give a cautious overall interpretation of results considering objectives, limitations, multiplicity of analyses, results from similar studies, and other relevant evidence	Sections 4.1–4.7 (associations interpreted as statistical, not causal; imprecision of the odds ratio explicitly discussed; findings compared with independent studies; clinical implications framed as preliminary and requiring confirmation in larger, longitudinal and multicentre studies before informing practice)
Generalisability	21	Discuss the generalisability (external validity) of the study results	Section 4.8 (predominantly urban, socio-culturally homogeneous sample; vulnerable populations not represented; findings to be interpreted within the Romanian healthcare context)
**OTHER INFORMATION**			
Funding	22	Give the source of funding and the role of the funders for the present study and, if applicable, for the original study on which the present article is based	Funding statement (this research received no external funding)

Note: An Explanation and Elaboration article discusses each checklist item and gives methodological background and published examples of transparent reporting. The STROBE checklist is best used in conjunction with this article (freely available at https://www.strobe-statement.org).
